# Acupuncture Reduces Apoptosis of Granulosa Cells in Rats with Premature Ovarian Failure Via Restoring the PI3K/Akt Signaling Pathway

**DOI:** 10.3390/ijms20246311

**Published:** 2019-12-13

**Authors:** Shiqi Wang, Shujun Lin, Mingmin Zhu, Chenglu Li, Shulian Chen, Liu Pu, Jihuan Lin, Luxi Cao, Yimin Zhang

**Affiliations:** 1 Department of Acupuncture, School of Traditional Chinese Medicine, Jinan University, Guangzhou 510632, China; sikeiwong@stu2018.jnu.edu.cn (S.W.); jun@stu2015.jnu.edu.cn (S.L.); jnuzmm@jnu.edu.cn (M.Z.); blutolee@stu2017.jnu.edu.cn (C.L.); emilychan@stu2016.jnu.edu.cn (S.C.); pl566199@stu2016.jnu.edu.cn (L.P.); linjh@stu2016.jnu.edu.cn (J.L.); 2 Formula-pattern Research Center, School of Traditional Chinese Medicine, Jinan University, Guangzhou 510632, China

**Keywords:** PI3K/Akt, premature ovarian failure, acupuncture, granulosa cells

## Abstract

Acupuncture is widely recognized as an effective therapy for premature ovarian failure (POF) in clinical, but information about its potential mechanisms is rarely explored. To investigate the mechanism, fifty SD female rats were randomly divided into normal group, POF group, POF+estradiol-valerate group (abbreviated as estradiol group), and POF+acupuncture group (abbreviated as acupuncture group). The estrous cycle of the rats was tracked by vaginal smears. Their ovaries morphology was observed by hematoxylin-eosin staining. The apoptotic level of granulosa cells was detected by in situ TUNEL fluorescence staining assay. Serum follicle-stimulating hormone (FSH) and estrogen (E2) levels were measured by enzyme-linked-immunosorbent-assay (ELISA). Protein and gene expression of PI3K, Akt, bcl-2, and bax were detected by Western blotting and qPCR. In the acupuncture and estradiol groups, compared with the POF group as controls, the apoptosis number of granulosa cells was significantly decreased (*p* < 0.05). FSH levels were decreased, while E2 levels were increased (*p* > 0.05). The gene and protein expression levels of PI3K, Akt, and bcl-2 were increased, while the expression levels of bax were decreased (*p* < 0.05), and the protein expression level of p-Akt increased. There was no significant difference between the acupuncture group and the estradiol group (*p* > 0.05). Acupuncture was able to regulate hormone levels in POF rats, up-regulate PI3K/Akt signaling pathway, and reduce the apoptosis of granulosa cells. This may be one of the mechanisms of acupuncture treating premature ovarian failure.

## 1. Introduction

Premature ovarian failure (POF) is commonly used to describe women under the age of 40, whose basic manifestations are amenorrhea, high gonadotropin, hypogonadism, and infertility [1]. Elevated serum follicle-stimulating hormone (FSH) and decreased estrogen (E2) levels are common features of POF [2]. The incidence rate has gradually increased from 1% [3], accompanied by a trend of rejuvenation. POF has serious effects on women’s physical and mental health [4], including psychological distress, infertility, osteoporosis, autoimmune diseases, ischemic heart disease, and increased risk of death [1]. Nowadays, the treatments of POF are hormone replacement therapy, ovulation induction therapy, ovarian transplantation, immunotherapy, ovulation induction therapy, and so on. Among them, estrogen drugs with good therapeutic effects, as common drugs, have large side effects, which can raise the recurrence rate after stopping and easily cause cancer, so they are not suitable as a long-term treatment method [5]. However, a large number of clinical studies have shown that we have used the acupuncture therapy, a unique treatment of traditional Chinese medicine, to treat POF, which makes the total effective rate of treatment reach more than 89%. It can regulate the function of the female reproductive endocrine system, meanwhile, it stimulates the development of follicles in the ovary to restore ovarian reserve function and promote ovulation [6,7]. According to the research of Han Zhang [8], POF female mice treated by electroacupuncture were mated with male mice, and the final result was that the number of litters in the electroacupuncture group increased compared with the model group (*p* < 0.05), which indicated that acupuncture has the effect of protecting fertility. Acupuncture not only has obvious therapeutic effects without the side effects of estrogen, but also has the advantages of simplicity, convenience, low cost, and efficiency [9]. Thus, acupuncture is more conducive to clinical promotion and application, but its mechanism of action is unclear and needs further exploration and interpretation.

The ovary is inseparable from women’s growth and development and directly affects their reproductive function. The number of primordial follicle pools in the ovary is fixed and cannot proliferate after birth. Simultaneously, the stillness, survival, and activation of follicles in the pool depends on the dynamic balance. This dynamic balance is regulated by many signaling molecules or pathways [10]. Abnormality of genetic factors, iatrogenic factors, autoimmune factors, congenital enzyme deficiency factors, infections and environmental factors, and idiopathic factors can break the follicular environment [11], which may cause follicles in the follicular pool to be inactivated or prematurely activated, even to degeneration and atresia at different stages of development. Finally, only a few follicles can complete development and ovulation [12], resulting in ovarian reserve functional failure and forming POF.

In recent years, experimental studies have found that granulosa cell apoptosis is the central link in the initiation of follicular atresia [13]. Granulose cells (GC) are generally multi-layered, closely linked to oocytes and located outside the zona pellucida. There is a complex connection mechanism between them, which is oocytes guiding the proliferation and differentiation of granulosa cells, and the corresponding granulosa cells providing key nutrients and signals for the maturation of oocytes [14]. To ensure the normal reserve function of the ovaries, the two cells must be interdependent and inseparable.

It is worth noting that when the ovarian reserve function gradually declines, hormones are involved in the apoptosis process of granulosa cells as an important influencing factor. FSH is an important hormone in ovarian development. Ovary development relies on a vital hormone FSH, which can combine with the specific receptors on ovarian granule cell membranes to activate those upstream protein kinases and GAB2. After that, the downstream target factors and the PI3k/akt pathway will be activated [15], or cell apoptosis will be slowed down [16]. FSH can also make induction of aromatase expression, regulates the secretion of estrogens and progesterone, and promote ovarian granulosa cell maturation [17]. E2 is a highly biologically active hormone in women that not only regulates gene transcription but also activates the PI3K signaling pathway in conjunction with estrogen receptor (ER) on the cell membrane [18]. By regulating hormone levels to up-regulate the expression of the PI3K/Akt signaling pathway, and improve follicular development and granulosa cell survival and proliferation, thereby restoring ovarian function, which may be one of the therapeutic directions of POF.

Hence, based on clinical research, it is important to carry out acupuncture treatment of POF on granulosa cell apoptosis.

## 2. Results

### 2.1. Acupuncture can Regulate and Restore the Normal Estrous Cycle Changes in POF Rats

**Figure 1:**
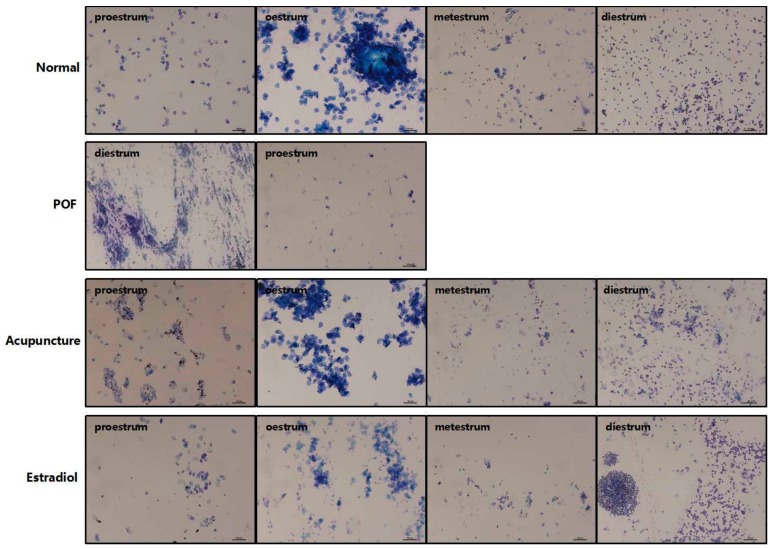
Effect of acupuncture on the estrous cycle in the premature ovarian failure (POF) model and the vaginal smears can be observed after methylene blue staining (×100 magnification. Scale bars: 10 μm.)

The estrous cycle of normal rats is 4–5 days. Vaginal smears at proestrus are mainly composed of large granulosa and plaque-like keratinocytes. White blood cells and middle layer cells are the main cells and have strong cytoplasmic staining, in the metestrus. After modeling, the rats in the POF model showed periodic disorders, such as prolonged cycle or cycle arrest, the number of exfoliated keratinized epithelial cells is sharped decrease, leukocytosis, intermediate cells reduced, and cell morphology atrophy. With nucleus loss, the cytoplasm of exfoliated cells are stained more lightly and the smears are accompanied by a large amount of mucus. Those suggesting successful modeling, most of the rats are in the diestrus or proestrus. After treatment, compared with the POF group, the estrous cycle of acupuncture group and estradiol group gradually returned to normal, whose reduction of vaginal exfoliated cells was improved, but the number of vaginal exfoliated cells was still less than that of the normal group (Figure 1).

### 2.2. Acupuncture Can Ameliorate Morphological Abnormalities and Apoptosis of Granulosa Cell in Ovarian Tissue of POF Rats

**Figure 2:**
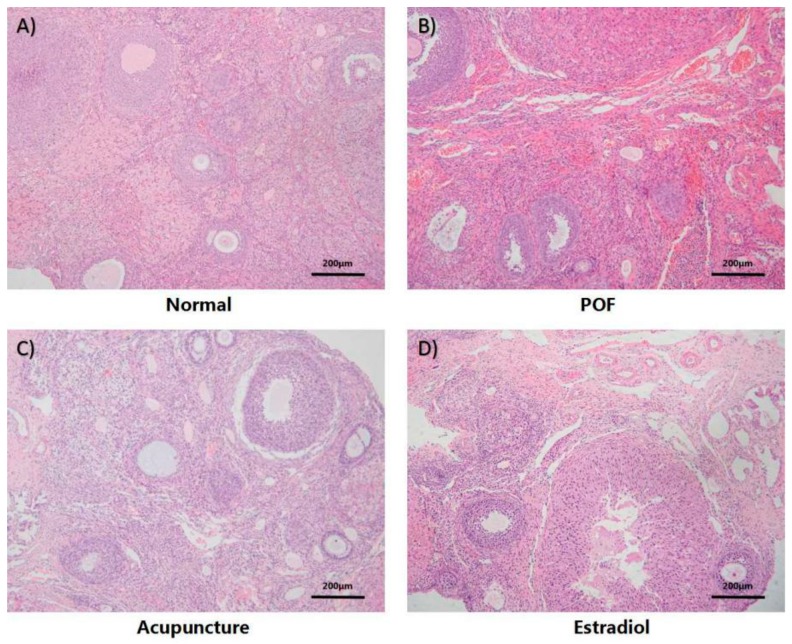
Effect of acupuncture on ovarian morphology and follicular development in the POF model, which were observed by the ovarian section after hematoxylin and eosin (HE) staining (×100 magnification. Scale bars: 200 μm). (**A**–**D**) are the representatives of the four groups respectively.

**Figure 3:**
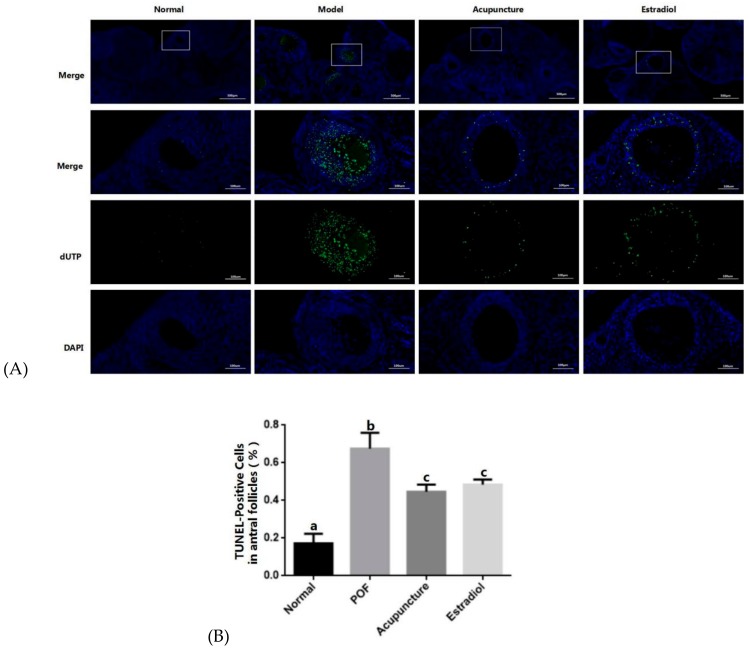
Effect of acupuncture on apoptosis of ovarian cells induced by cyclophosphamide. In situ TUNEL fluorescence staining was used to analyze apoptosis. In the TUNEL assay, TUNEL-positive nuclear (apoptotic) cells were stained with green (**A**), and the white boxes are the representatives of random spots we have selected in groups. The TUNEL-positive granulosa cells and total granulosa cells in the antral follicles in random spots were counted. The percentage of the number of TUNEL-positive granule cells (%) among the four groups were compared (*n* = 10) (**B**). Statistical significance: Different letters represent statistically significant differences between groups (a vs. b: *p* < 0.05, b vs. c: *p* < 0.05), whereas means that share the same letter do not differ significantly (*p* > 0.05).

Compared with the normal group, the ovarian morphology of the POF rats was atrophied to a certain extent, the ovarian cortex gets thickened and has a disordered structure. Follicular vacuole-like changes in all stages of the follicles were obvious loosely arranged, increased atresia follicles, interstitial fibrosis, vascular congestion, decreased mature follicles, and increased secondary corpus luteum (Figure 2A,B). For granulosa cells mainly attached to the oocyte clear zona pellucida, the positive rate of apoptosis in the POF group was significantly higher than the normal group in the follicular cavity (*p* > 0.05). Compared with the POF group, acupuncture, and the estradiol group improved similarly, the ovarian morphology became normal, the oocyte morphology was improved compared with the POF group, and the ovarian primordial follicles, primary follicles, and mature follicles increased;(Figure 2C,D) granulosa cell apoptosis level is reduced, and the decrease in the number of apoptosis in the acupuncture group was lower than the estradiol group, but it was not statistically significant (*p* > 0.05) (Figure 3).

### 2.3. Acupuncture Can Up-Regulate Serum E2 Levels in POF Rats and Down-Regulate Serum FSH Levels

**Figure 4:**
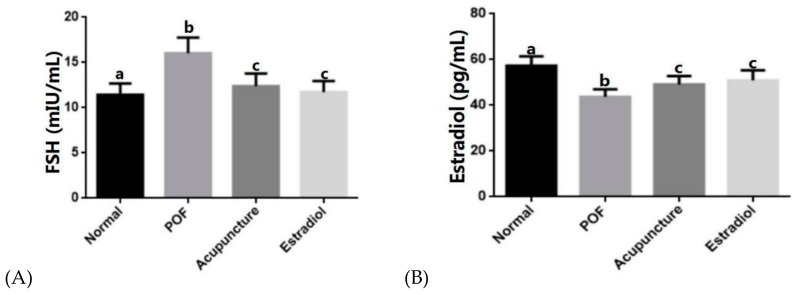
Protective effects of acupuncture on the ovarian E2. The serum follicle stimulating hormone (FSH) (**A**), E2 (**B**) levels were tested in four groups. All data are shown as the mean ± s.d. Statistical significance: Different letters represent statistically significant differences between groups (a vs. b: *p* < 0.05, b vs. c: *p* < 0.05), whereas means that share the same letter do not differ significantly (*p* > 0.05).

Compared with the normal group, serum E2 levels in the POF group were decreased (*p* < 0.05) and FSH levels were increased (*p* < 0.05). After treatment with drugs or acupuncture, compared with the POF group, the level of the E2 in the acupuncture group and estradiol group increased, and the level of FSH decreased (*p* < 0.05). There was no statistical difference between the two groups (*p* > 0.05) (Figure 4).

### 2.4. Acupuncture Can Regulate the Expression of PI3K/AKT Pathway as well as the Gene and Protein Expression of Bcl-2 and Bax Apoptosis in POF Rat Ovary

**Figure 5:**
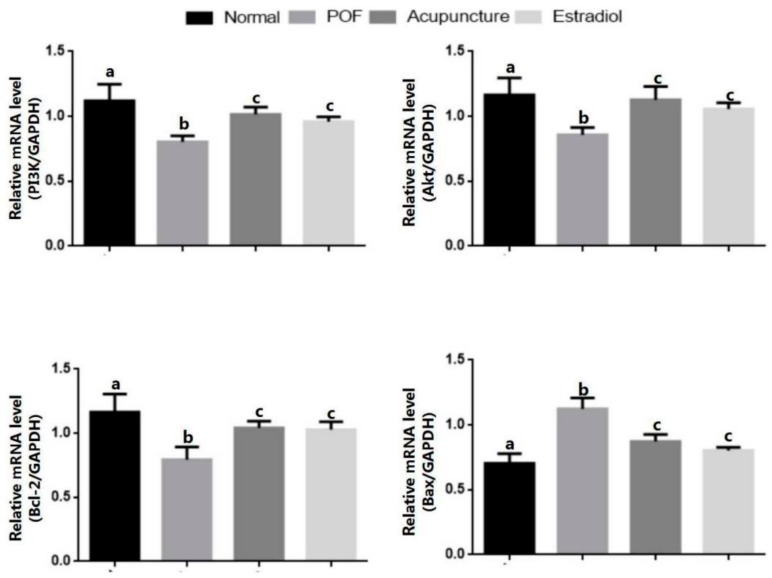
Effect of acupuncture on the expression of the gene associated with apoptosis in ovarian tissues. The gene expression levels of *PI3K, Akt, bcl-2,* and *bax* in ovarian tissues were detected by using Q-PCR, which were analyzed quantitatively. Data are shown as the mean ± s.d. Statistical significance: Different letters represent statistically significant differences between groups (a vs. b: *p* < 0.05, b vs. c: *p* < 0.05), whereas means that share the same letter do not differ significantly (*p* > 0.05).

**Figure 6:**
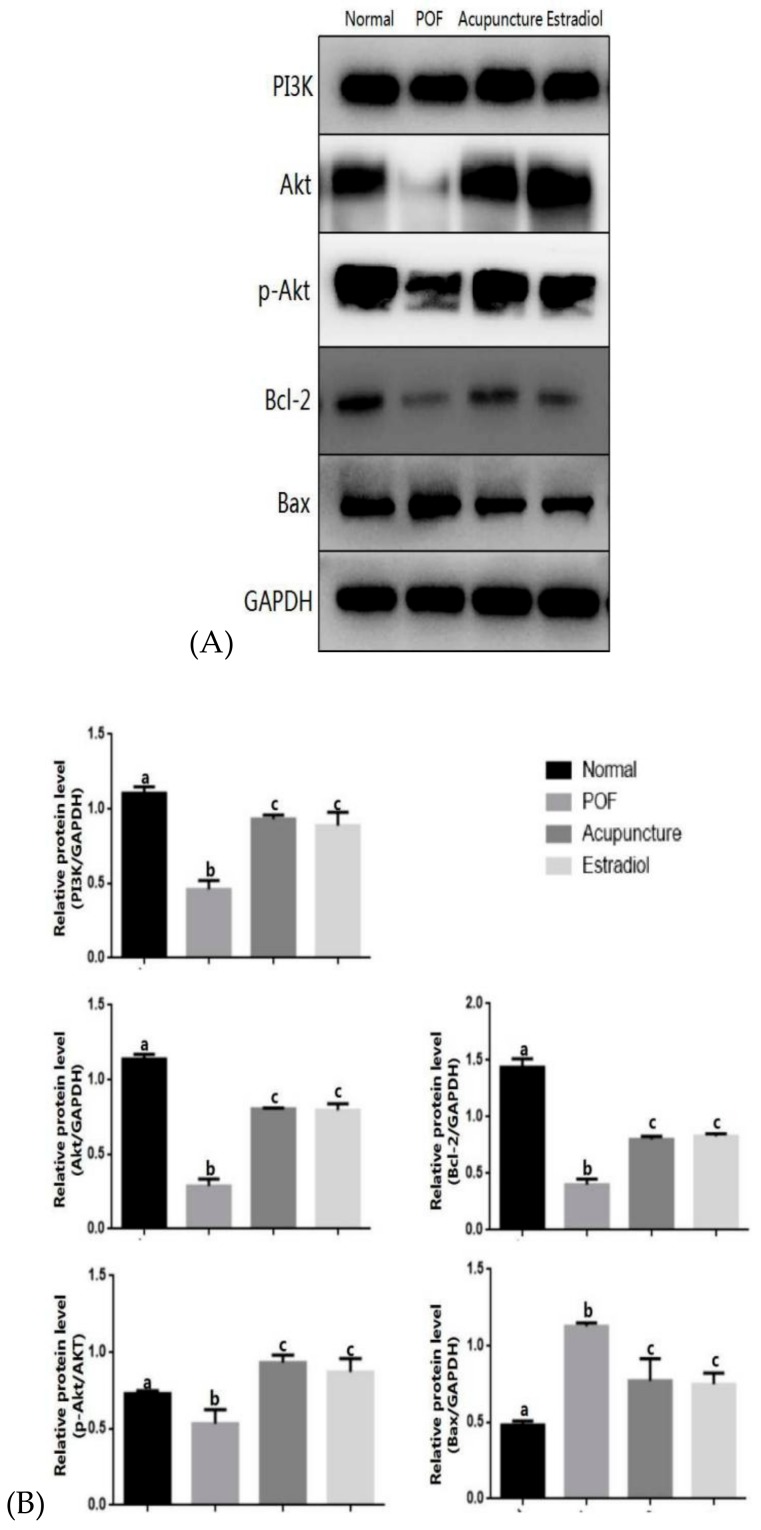
Effect of acupuncture on the expression of the protein associated with apoptosis in ovarian tissues. The protein expression levels PI3K, Akt, bcl-2, and bax in ovarian tissues were detected by using Western blotting (**A**), which were quantitatively analyzed (**B**). Data are shown as the mean ± s.d. Statistical significance: Different letters represent statistically significant differences between groups (a vs. b: *p* < 0.05, b vs. c: *p* < 0.05), whereas means that share the same letter do not differ significantly (*p* > 0.05).

Compared with the normal group, the gene and protein expression levels of PI3K, Akt, and bcl-2 in the POF group were decreased (*p* < 0.05) and bax was increased (*p* < 0.05). After drug-supplemented estrogen therapy or acupuncture treatment, the gene and protein expression levels of PI3K, Akt, and bcl-2 in the acupuncture group and estradiol group were increased compared with model group (*p* < 0.05), and the bax was decreased (*p* < 0.05). In addition, the protein expression level of p-Akt in the POF group showed a downward trend compared with the normal group, which in the acupuncture group and estradiol group increased. There was no statistical difference between the two groups (*p* > 0.05) (Figure 5 and Figure 6).

## 3. Discussion

Premature ovarian failure is an endocrine-altering disease caused by a combination of factors. It is characterized by decreased estrogen, elevated gonadotropin, increased granulosa cell apoptosis, poor nutrition and survival environment of oocytes, and follicles atresia. Ultimately, it leads to a decline in ovarian storage function. Therefore, the reduction of effective follicles is the key to the pathogenesis of premature ovarian failure. Maintaining the normal survival of primordial follicles is an essential condition for preserving normal ovarian function [19]. In physiological conditions, the phosphatidylinositol 3-kinase (PI3K) signaling pathway plays an important role in the regulation of granulosa cell activation, development, and proliferation with hormone induction, which is also critical for the growth and maturation of activated follicles. Specific PI3K can successfully catalyze the formation of PIP3 by PIP2 on the inner surface of the membrane [20]. PIP3 shares homology with the PH domain, whose special structural protein can accumulate under the cell membrane to activate the Pdk1 factor, and then serine kinase could be activated. Phosphorylated Akt can activate the protein of downstream Bcl-2 gene family to phosphorylate and regulate the translation of other protein and gene transcription. It flexibly regulates the quiescence, activation, and development of primordial follicles [21]. Abnormal expression of various signaling molecules in the PI3K pathway in human oocytes may lead to defects in the survival and development of primordial follicles [22], which arouses POF. Here we mainly discuss the close relationship among the normal function of the ovary, granulosa cells, and the hormone-induced PI3K/Akt signaling pathway.

The international standard specification chemotherapy drug cyclophosphamide was used to make models in this experiment. Cyclophosphamide is a commonly used anti-tumor drug for clinical treatment, which has significant immunosuppressive effects and cytotoxic effects [23]. The drug inhibits ovarian function and destroys follicles in the ovary, which in turn affects fertility [24] and was usually used to make POF rat models.

After intraperitoneal injection of cyclophosphamide, the ovaries of the rats were damaged by chemotherapeutic drugs. The granulosa cells of the follicles at the development stage were killed a lot, and the serum level of E2 secretion decreased while the level of FSH increasd [25]. Elevated levels of serum FSH may indicate the degree of ovarian toxicity induced by cyclophosphamide [26], which combined with E2 levels will be better as an early predictor of ovarian reserve [25]. FSH can promote the phosphorylation of Akt in ovarian granulosa cells. FSH can promote the phosphorylation of Akt in ovarian granulosa cells. The Activated Akt, which is phosphorylated Akt (p-Akt) causes knock-on effects at all levels of signaling pathways [27], promotes protein synthesis and matures ovarian granulosa cells to produce more estradiol. However, due to the massive destruction of granulosa cells, the expression of FSH receptors in cells reduced, and the normal physiological effects of FSH cannot be exerted [14]. At the same time, E2 also loses a large number of synthetic sites. In addition, studies have shown that E2 can inhibit the secretion of FSH from the pituitary gland under the feedback of the pituitary-ovary axis [28], so the FSH stress is elevated in the low level of E2.

After the treatment finished, the number of mature follicles in the acupuncture group and estradiol group were elevated, and the level of estrogen secretion increased. The reason is that the estrogen ligand binding to the G-protein coupled receptor (GPR30) on the cell surface increases, thereby activating the intracellular PI3K/Akt signaling pathway [29]. The PI3K/AKT pathway serves as a bridge between extracellular signals and cellular responses, downstreaming signaling molecules, and playing an important role in regulating apoptosis and glycolipid metabolism under the influence of upstream signaling molecules [30]. PI3K triggers Akt activation. Phospho-Akt phosphorylates a range of downstream target proteins, including anti-proliferative and anti-apoptotic FOXO3a, members of the Bcl-2 family, BAD involved in the mitochondrial apoptosis pathway, mTOR controlling biosynthesis of protein and regulating cell growth, and p27 maintaining the original follicle reserve [31]. Akt can also phosphorylate Bax (Ser184) to prevent the formation of homodimers and block the pro-apoptotic effect of Bax [32]. Many functions of the PI3K/Akt signaling pathway are mainly accomplished by p-Akt phosphorylating many effectors of downstream [30]. The pathway could promote gene transcription and translation of anti-apoptotic gene proteins, including Bcl-2 gene family, and play an important role in promoting cell growth and inhibiting apoptosis [33]. However, the serum levels of estrogen and the expression of the PI3K/Akt pathway in the POF group remained low, and the anti-apoptotic gene protein could not be activated while the activity of the apoptotic protein increased. The apoptotic protein bax promotes apoptosis. When the bax protein expression was elevated, bax could bind to each other to form homodimers, inducing granulosa cell apoptosis. Meanwhile, Bcl-2 protein expression was reduced, so bcl-2 was unable to connect with bax forming heterodimers to regulate apoptosis, which resulted in an imbalance in the Bcl-2/bax ratio, and accelerated granulosa cell apoptosis [34]. FSH and estrogen interact by activating the PI3K/Akt signaling pathway and its downstream signaling pathways to achieve optimal regulation of the hormones [35], which is of great significance for the early detection, early treatment, and restoration of ovarian function in POF.

Our experimental results showed that in the early stage of POF formation, the efficacy of acupuncture is equivalent to that of the drug. In addition to gradually recovering the estrous cycle, regulating serum sex hormone levels and improving morphological abnormalities of ovarian tissue, the basic conditions in the acupuncture group improved significantly, including hair color, body weight, appetite, and the mental state of the rats. Acupuncture can up-regulate serum estrogen levels in the POF model rats and restore the PI3K/Akt pathway to up-regulate the expression of the *bcl-2* gene and protein in granulosa cells with the expression of bax decreasing. Then the bcl-2/bax heterodimer whose ratio is increased is more stable than the bax homodimer. At last, the follicular atresia is reduced and the apoptosis of granulosa cells is effectively inhibited, which is beneficial to provide nutrient signals for follicles by granulosa cells and restore ovarian function. It may be one of the mechanisms of acupuncture treating premature ovarian failure. What is lacking in this experiment is to evaluate the fertility function of POF rats after acupuncture treatment. Later, an experiment can be added to observe the litter size of the POF rats after treatment. The efficacy of acupuncture on POF can be judged macroscopically, which will be more clinically realistically significant.

## 4. Materials and Methods

### 4.1. Experimental Animal

SD female rats (*n* = 50, 4–6 weeks old, SPF grade, weigh 220 ± 20 g) were purchased from Jinan Pengyue Experimental Animal Breeding Co., Ltd. The animal certificate number is SCXK (Lu)20140007. Adequate food and tap water were freely available to all animals, who were observed for 1 week of isolation before random experimentation. All experimental personnel conducted experiments under the supervision and guidance of the Laboratory Animal Ethics Committee of Jinan University, whose license number is SCXK (Yue)20170174, and the approval number of our experiments is 2018118-07. All procedures were in compliance with the Statute on the Administration of Laboratory Animal approved by China’s Council (November 1988).

### 4.2. Modeling and GROUPING

After a week of adaptive feeding, the estrous cycle of each rat was tracked by vaginal smears for 7 days. Rats with a normal estrous cycle were included in the experiment. 10 rats were randomly selected as the normal group according to the random number table. The remaining 40 rats were used for modeling and were intraperitoneally injected with cyclophosphamide (C0768-1G, Sigma, Burlington, MA, USA) for the first day 50 mg/kg dose, and 8 mg/kg dose continuously for 14 days to build the POF models. During the modeling process, the rats were vaginally smeared at a fixed time each day, whose ectopic cycle changes were observed under the microscope (ECLIPSE TS100-F. Nikon, Tokyo, Japan). Fifteen days later, female rats with successful modeling were randomly divided into the POF group (*n* = 14), a POF+acupuncture group (abbreviated as acupuncture group) group (*n* = 13), and a POF+estradiol-valerate group (abbreviated as estradiol group) group (*n* = 13).

### 4.3. Animal Treatment

The acupuncture and estradiol groups started treatment after successful modeling. The acupuncture points were selected on the basis of the acupoint pattern of the rats [36], including Baihui (DU 20), Guanyuan (RN 4), Zigong (EX-CA 1), bilateral collateral (LI 4), bilateral Taichong (LR 3), and bilateral Sanyinjiao (SP 6). After rats being fixed, sterile acupuncture needles (diameter: 0.18 mm, length: 15 mm, made by Suzhou Shenlong Medical Instrument Co., Ltd., Suzhou, China) were inserted perpendicularly into the abovementioned acupuncture points, which depth was 2 mm. Then the needle would be retained for 15 min, and each needle was twirled with an even reinforcing-reducing method every 5 min. The estradiol group was also fixed and treated daily with estradiol valerate for intragastric administration. The equivalent dose of the drug was calculated according to the body surface area of the human and the animal, in which the daily dose of the rat was equivalent to 6.3 times of an adult’s. So the intragastric dose of the rat was 1 mg/60 kg × 6.3 Bujiale (estradiol valerate, J20171038, Delpharm Lille S.A.S., Lys Lez Lannoy, France) at a concentration of 1 mL/100 g every day. Both the estradiol group and the acupuncture group were treated for 3 weeks. The POF and the normal groups received no treatment except for being fixed for 15 min per day. All rats fixed with a unified fixed device during the experiment. First, the rat was placed in a prone position on the platform of the fixed frame. Then, the rat’s head was exposed in the middle of the front iron pillars, its tail was put between the end iron pillars, the limbs were exposed to the outside of the pillars, and its body was tied to a fixed frame with matching straps.

### 4.4. Tissue Collecting

We draw the materials from the rats within 24 h after the end of the treatment period. First, the rats were anesthetized by intraperitoneal injection of 2% pentobarbital (0.15 mL/100 g) and fixed on the sampling table. Second, the abdominal skin of the rat was cut open to fully expose the abdominal cavity and blood was collected from the abdominal aorta after blunt dissection. The ovaries and uterus were removed and weighed, then half of the tissue was snap-frozen and stored at −80 °C for further analysis, another part was fixed in 4% paraformaldehyde (P1110-2, Nocon Bio Company, Guangzhou, China).

### 4.5. Indicator Detection

#### 4.5.1. Determination of Estrus Cycle

Vaginal smears were obtained to monitor the estrus cycle. Following the collection of the smears by swabbing the vagina of rat, the morphology of the obtained epithelial cells were smeared and analyzed under a light microscope (ECLIPSE TS100-F. Nikon, Tokyo, Japan) after the smears were stained by methylene blue staining (0.1%, Solaibao Technology Co., Ltd. Beijing, China) and air-dried.

#### 4.5.2. Hematoxylin and Eosin Staining

Ovary tissues were fixed with 4% polyformaldehyde (P1110-2, Nocon Bio Company, Guangzhou) for 24 h, and dehydrated. Subsequently, the tissues were embedded in paraffin and cut into 5-µm-thick sections to be placed on numbered glass slides. To investigate the distribution of morphological changes of ovarian tissue in rats, the prepared tissues were stained with hematoxylin and eosin (HE) (Servicebio, Wuhan, China), photographed, and analyzed by using light microscope (ECLIPSE TS100-F. Nikon, Japan).

#### 4.5.3. In Situ TUNEL Fluorescence Staining Assay

The steps of fixation, dehydration, paraffin embedding, and sectioning are the same as above. The terminal deoxynucleotidyl transferase (TdT)-mediated deoxyuridine triphosphate (dUTP) nick end labeling (TUNEL) assay was performed according to the manufacturer’s instructions. The paraffin sections were dewaxed at 60 °C for 60 min and rehydrated with ethanol (100%, 90%, 80%, and 75%) (Sinopharm Chemical Reagent Co., Ltd. Shanghai, China) in a step by step manner. The slides were washed thrice with PBS (pH = 7.4, G0002, Servicebio, Wuhan, China) for 5 min and incubated with proteinase K (G1205, Servicebio, Wuhan, China) at 37 °C for 25 min. After being dried, the slide had enough rupture solution added, which was incubated at room temperature for 20 min, and washed thrice with PBS (pH = 7.4, G0002, Servicebio, Wuhan, China) for 5 min again. Suitable microlitre TdT and dUTP enzyme reaction mixture (TUNEL Kit, 11684817910, Roche, Basel, Switzerland) was added to covering the samples, and the whole setting was incubated for 2 h at 37 °C in a humidified atmosphere in the dark and washed thrice in PBS (pH = 7.4, G0002, Servicebio, Wuhan, China) for 5 min. Then, the cell nucleus was dyed with DAPI (G1012, Servicebio, Wuhan, China) in the dark for 10 min. The sections were observed with fluorescence microscopy (ECLIPSE C1. Nikon, Japan) and the apoptotic cells in the ovary were stained green.

To avoid histological differences among samples, five random spots per slice (three slices per rat, ten rats per group, *n* = 10) were detected. Totally, 150 random spots (5 × 3 × 10 = 150) per group were checked. At each random spot, the TUNEL-positive granulosa cells and the total granulosa cells in the antral follicles were counted. The percentage of TUNEL-positive granulosa cells (%) in the antral follicles was analyzed by the Image Pro Plus 6.0 software.

#### 4.5.4. Enzyme-Linked Immunosorbent Assay (ELISA)

Blood samples were obtained from rats models by abdominal aortic blood collection to detect the levels of serum E2 and FSH. All blood samples were allowed to stand at room temperature for 1 h, then those samples were centrifuged at 3000 r/min for 15 min to collect the supernatant. Finally, the concentration of the hormone was measured by ELISA kits (JYM0608Ra and JYM0597Ra, Jiyinmei Technology Co., Ltd. Wuhan, China). Quantitative determination of the hormone concentrations was performed according to the manufacturer’s protocols.

#### 4.5.5. RT-qPCR

mRNA expression of PI3K, Akt, bcl-2, and bax was measured using quantitative real-time reverse transcriptase PCR (RT-qPCR). Total RNA was isolated from the ovarian tissue of rats using RNAiso Plus (9108, TaKaRa, Dalian, China) according to the manufacturer’s instructions. RNA concentration and purity were measured using a NanoDrop 1000 (Thermo Scientific, Rockford, Illinois, USA). Total RNA diluted to a standard concentration of 500 ng/mL was mixed with the PrimeScript RT-reagent kit (RR037A, TaKaRa, Dalian, China) to form an RT reaction solution, and the reverse transcription reaction was performed by Mastercycler nexus PCR instrument after gentle mixing the mixture to synthesize cDNA. Then RT-qPCR was performed using a CFX Connect Real-Time PCR Detection System (BIO-RAD, Hercules, California, USA) with a PCR reaction solution composed of reverse transcriptional production cDNA and SYBR Premix EX Taq II (RR820A, TaKaRa, Dalian, China) according to the manufacturer’s instructions. Primers were designed and synthesized by Ruizhen Biotechnology Co. (Shanghai, China). The following primers were used: rat PI3K sense: 5′-CTTGCCTCCATTCACCACCTCT-3′, antisense: 5′-GCCTCTAATCTTCTCCCTCTCCTTC-3′; rat Akt sense: 5′-TGTCTCGTGAGCGCGTGTTT-3′, antisense: 5′-CCGTTATCTTGATGATGTGCCCGTC-3′; rat bcl-2 sense: 5′-ACTTCTCTCGTCGCTACCGTCG-3′, antisense: 5′-CCCTGAAGAGTTCCTCCACCACC-3′; rat bax sense: 5′-CATGAAGACAGGGGCCTTTTTG-3′, antisense: 5′-TCAGCTTCTTGGTGGATGCGTC-3′; rat GAPDH sense: 5′-TCTCTGCTCCTCCCTGTTC-3′, antisense: 5′-ACACCGACCTTCACCATCT-3′. After the polymerase chain reaction, the expression level of related genes was analyzed by the relative quantitative method and relative standard curve, and each sample was detected three times with an average of three times.

#### 4.5.6. Western Blot

Proteins were extracted from the ovarian tissues of rats and then quantified using BCA protein assay kits (BB18091, BestBio Science, Shanghai, China). An equal amount of protein (20 mg/lane) was conducted electrophoresis by using an electrophoresis apparatus (BIO-RAD, USA) on SDS-PAGE gel (P0012AC, Beyotime Biotechnology, Shanghai, China). Then the selected target gel band was transferred to PVDF membranes (IPVH00010, Millipore, Darmstadt, Germany) by Trans-Blot Turbo Transfer System (BIO-RAD, Hercules, CA, USA). The membranes were blocked in 5% skimmed milk (FD0080, Fdbio science, Hangzhou, China) at room temperature for 1 h and then incubated overnight at 4 °C with the following primary antibodies: rabbit anti-PI3K monoclonal antibody (1:1000, #4257, Cell Signaling Technology, Beverly, MA, USA); rabbit anti-Akt monoclonal/ antibody (1:1000, #4691, Cell Signaling Technology, Beverly, MA, USA); rabbit anti-phospho-Akt monoclonal antibody (1:1000, AF3261, Affinity Biosciences, Wembley, Middlesex, UK); rabbit anti-bax monoclonal antibody (1:1000, #14796, Cell Signaling Technology, Beverly, MA, USA); mouse anti-bcl-2 monoclonal antibody (1:1000, abs132008, Absin, Shanghai, China); mouse anti-GAPDH monoclonal antibody (1:5000, #A01020, Abbkine, Wuhan, China). The membranes were washed with Tris-buffered saline and Tween 20 (TBST) prior to incubation with corresponding goat anti-rabbit IgG(H+L) secondary antibody (1:5000, #A21020, Abbkine, Wuhan, China) or goat anti-mouse IgG(H+L) secondary antibody (1:5000, #7076, Cell Signaling Technology, USA) for 1 h. Finally, protein bands were detected by electrochemiluminescence kit (AC36131, Bioword, USA) and analyzed by GE Image Quant LAS 4000 mini imaging analysis software (BIO-RAD, Hercules, CA, USA) after ChemiDoc Imaging System (BIO-RAD, USA) imaging according to the manufacturer’s instructions.

#### 4.5.7. Statistical Analysis

Statistical analysis was performed using SPSS software (V13.0, IBM, Chicago, USA). Data are presented as mean ± SD. Groups were compared using one-way ANOVA. When the variance was homogeneous, S-N-K (Student–Newman–Keuls) method was used to compare the two groups; otherwise, Tamhane’s T2 method was used when the variance is uneven. A *p*-value of < 0.05 was considered to be statistically significant.

## 5. Conclusions

In summary, the study explored the effect and mechanism of acupuncture treating POF. Acupuncture can regulate the hormone level, up-regulate the expression of the PI3K/Akt signaling pathway and bcl-2, which is an anti-apoptotic gene. Acupuncture can also lower the expression of apoptotic gene bax and reduce granulosa cell apoptosis and follicular atresia. This experiment provides a reliable basis for clinical treatment of POF which is conducive to facilitating the promotion and application of acupuncture in the clinic.
